# PD-1 blockade counteracts post–COVID-19 immune abnormalities and stimulates the anti–SARS-CoV-2 immune response

**DOI:** 10.1172/jci.insight.146701

**Published:** 2021-12-22

**Authors:** Cristian Loretelli, Ahmed Abdelsalam, Francesca D’Addio, Moufida Ben Nasr, Emma Assi, Vera Usuelli, Anna Maestroni, Andy Joe Seelam, Elio Ippolito, Stefania Di Maggio, Lara Loreggian, Dejan Radovanovic, Claudia Vanetti, Jun Yang, Basset El Essawy, Antonio Rossi, Ida Pastore, Laura Montefusco, Maria Elena Lunati, Andrea M. Bolla, Mara Biasin, Spinello Antinori, Pierachille Santus, Agostino Riva, Gian Vincenzo Zuccotti, Massimo Galli, Stefano Rusconi, Paolo Fiorina

**Affiliations:** 1 International Center for T1D, Pediatric Clinical Research Center Romeo ed Enrica Invernizzi, Department of Biomedical and Clinical Sciences “L. Sacco”, University of Milan, Milan, Italy.; 2 Nephrology Division, Boston Children’s Hospital, Harvard Medical School, Boston, Massachusetts, USA.; 3 Division of Respiratory Diseases, ASST Fatebenefratelli Sacco, Milan, Italy.; 4 Department of Biomedical and Clinical Sciences “L. Sacco”, University of Milan, Milan, Italy.; 5 Institute of Organ Transplantation, Tongji Hospital and Medical College, Huazhong University of Science and Technology, Wuhan, China.; 6 Medicine, Al-Azhar University, Cairo, Egypt.; 7 Transplantation Research Center, Nephrology Division, Brigham and Women’s Hospital, Boston, Massachusetts, USA.; 8 Endocrinology Division, ASST Fatebenefratelli Sacco, Milan, Italy.; 9 Division of Tropical Diseases and; 10 Division of Infectious Diseases, ASST Fatebenefratelli Sacco, Milan, Italy.; 11 Pediatric Clinical Research Center Romeo ed Enrica Invernizzi, Department of Biomedical and Clinical Sciences “L. Sacco”, University of Milan, Milan, Italy.; 12 Department of Pediatrics, Buzzi Children’s Hospital, Milan, Italy.; 13 Infectious Diseases Unit, Legnano General Hospital, ASST Ovest Milanese, Legnano, Italy.

**Keywords:** COVID-19, Immunology, Anergy, Immunotherapy

## Abstract

A substantial proportion of patients who have recovered from coronavirus disease-2019 (COVID-19) experience COVID-19–related symptoms even months after hospital discharge. We extensively immunologically characterized patients who recovered from COVID-19. In these patients, T cells were exhausted, with increased PD-1^+^ T cells, as compared with healthy controls. Plasma levels of IL-1**β**, IL-1RA, and IL-8, among others, were also increased in patients who recovered from COVID-19. This altered immunophenotype was mirrored by a reduced ex vivo T cell response to both nonspecific and specific stimulation, revealing a dysfunctional status of T cells, including a poor response to SARS-CoV-2 antigens. Altered levels of plasma soluble PD-L1, as well as of *PD1* promoter methylation and *PD1*-targeting miR–15-5p, in CD8^+^ T cells were also observed, suggesting abnormal function of the PD-1/PD-L1 immune checkpoint axis. Notably, ex vivo blockade of PD-1 nearly normalized the aforementioned immunophenotype and restored T cell function, reverting the observed post–COVID-19 immune abnormalities; indeed, we also noted an increased T cell–mediated response to SARS-CoV-2 peptides. Finally, in a neutralization assay, PD-1 blockade did not alter the ability of T cells to neutralize SARS-CoV-2 spike pseudotyped lentivirus infection. Immune checkpoint blockade ameliorates post–COVID-19 immune abnormalities and stimulates an anti–SARS-CoV-2 immune response.

## Graphical abstract

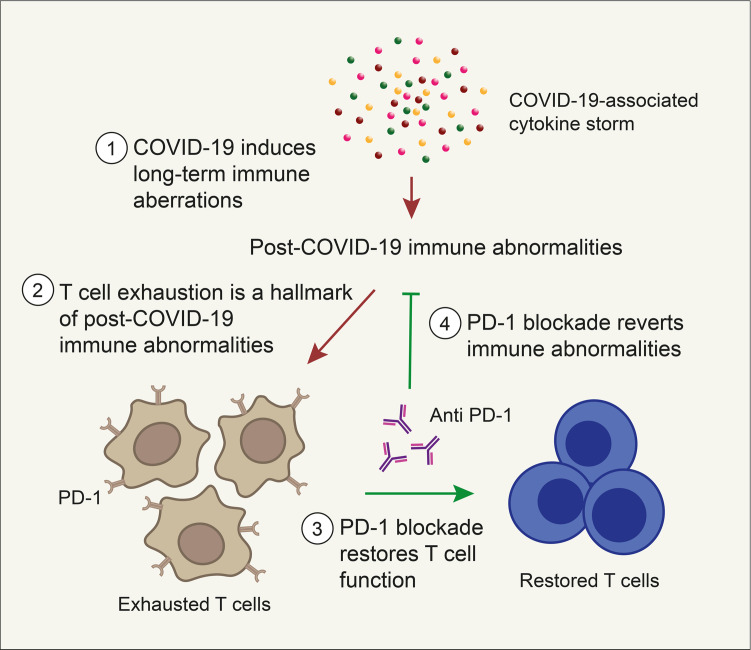

## Introduction

A large proportion of patients discharged after being hospitalized for COVID-19 experience the persistence of COVID-19–related symptoms (1–5), a condition defined as “long COVID” (6). During the acute phase of COVID-19, a variety of immune alterations are evident, including lymphopenia and proinflammatory cytokine storm (7–14). These immune disorders denote a broad functional impairment occurring in both the innate and adaptive compartments of the immune system that may also affect the ability to counteract SARS-CoV-2 infection. Interestingly, increasing evidence suggests that phenotypic and functional alterations of the immune system persist long after recovery from COVID-19 (15–17). These immune abnormalities may dampen an efficient immune response against viral reinfections and may overall impair a person’s ability to fight pathogens (18–20). The urgent need for an effective cure for COVID-19 has prompted the use of therapeutic strategies and accelerated the development of effective vaccines (21–37). Indeed, the success of widespread vaccination has been associated with reduced hospitalization and reinfection (36, 37). Designing pharmacological strategies to stimulate the immune system may be a valuable tool to abrogate or reduce over time the long-term sequelae of COVID-19–related symptoms. In this report, we characterize the immune profile of patients who eventually recovered form COVID-19 and describe a potentially novel strategy capable of reverting the extensive immune abnormalities observed; indeed, we also noted a PD-1 blockade–dependent enhancement of anti–SARS-CoV-2 immune response. A method to elicit a successful reversal of post–COVID-19 immune abnormalities may be necessary as long-term COVID-19 symptoms cause, in some cases, serious afflictions, and this strategy could therefore be significantly clinically relevant.

## Results

### Immune signature of patients who recovered from COVID-19.

With the aim of providing a comprehensive description of the immune signature of patients who recovered from COVID-19, we first conducted an extensive immunophenotyping analysis of cells obtained from healthy controls and patients, whose main clinical characteristics are reported in Table 1. Among lymphocytes, CD19^+^ B cells were slightly decreased in patients who recovered from COVID-19 as compared with healthy controls, while an increase in CD8^+^ T cells was evident in patients who recovered from COVID-19 as compared both with those in the acute phase and with healthy controls (Supplemental Figure 1A; supplemental material available online with this article; https://doi.org/10.1172/jci.insight.146701DS1). Patients who recovered from COVID-19 also showed a higher proportion of effector CD8^+^ T cells (CD45RO^–^CD62L^–^), while memory T cells, either effector or central (CD45RO^+^CD62L^–^ or CD45RO^+^CD62L^+^), were slightly altered among groups (Supplemental Figure 1, A and B). Furthermore, while the proportion of CD40L-expressing CD4^+^ T cells and that of ICOS-expressing CD8^+^ T cells was decreased in patients who recovered from COVID-19 as compared with patients with COVID-19 (Table 2), the fraction of OX40^+^ and GITR^+^ CD8^+^ T cells of patients who recovered from COVID-19 exceeded that of healthy controls, with the latter marker being the highest observed among all groups (Table 2).

**Figure 1:**
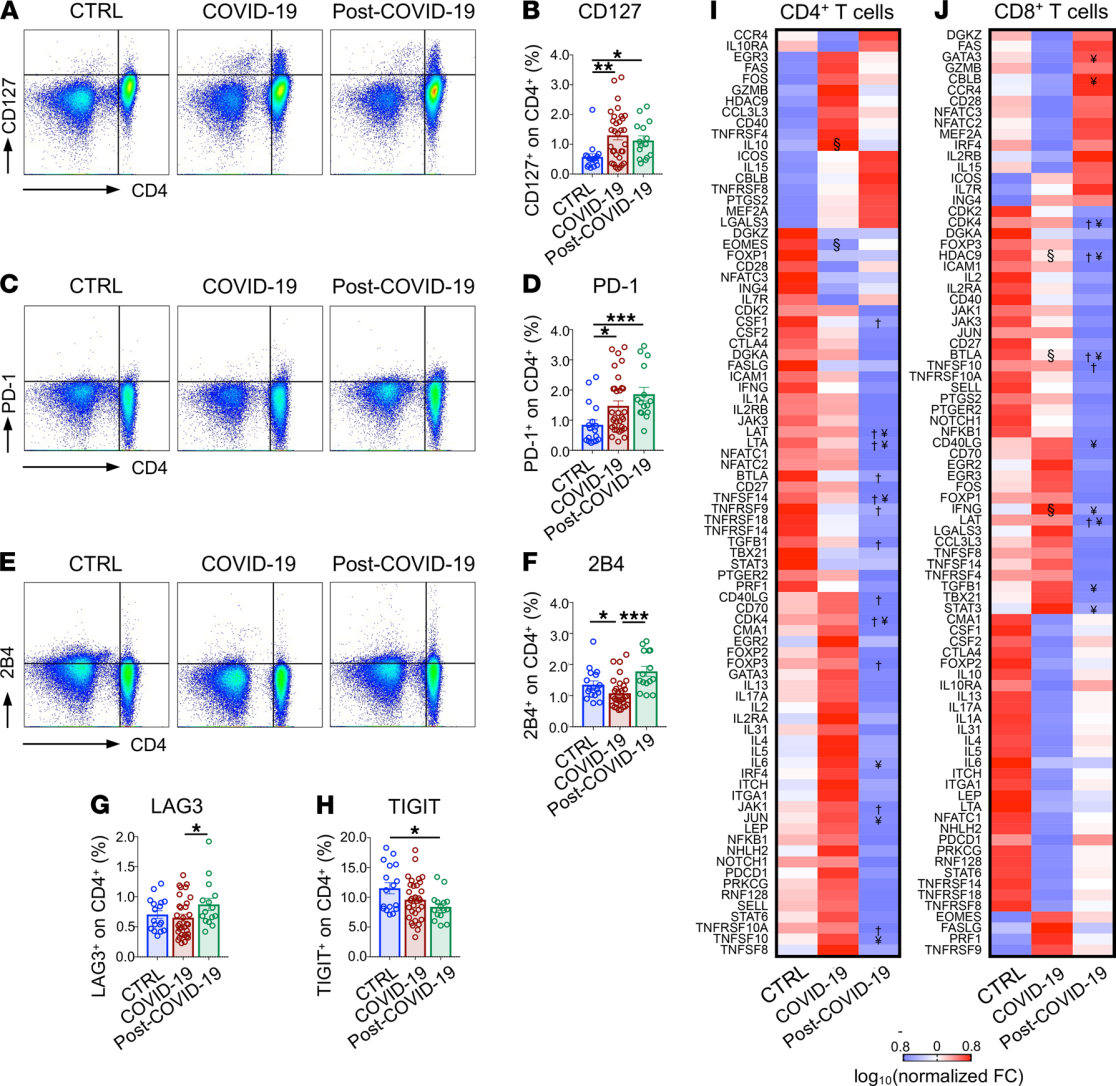
Immune signature of patients with COVID-19 and post–COVID-19 as compared with healthy controls. (**A**–**H**) Dot plot representations (**A**, **C**, and **E**) and bar graphs (**B**, **D**, and **F**–**H**) depicting the percentage of CD127^+^, PD-1^+^, 2B4^+^, LAG3^+^, and TIGIT^+^ CD4^+^ T cells as assessed by flow cytometric analysis in the same patient groups. (**I** and **J**) Heatmap representation of exhaustion marker transcriptomic profiling of isolated CD4^+^ (**I**) and CD8^+^ (**J**) T cells isolated from patients with COVID-19 (*n* = 3), from those who recovered from COVID-19 (*n* = 3), and in healthy controls (*n* = 3). Data in all panels are reported as mean ± SEM, unless otherwise reported. §, COVID-19 versus CTRL; †, post–COVID-19 versus CTRL; ¥, post–COVID-19 versus COVID-19; *P < 0.05, **P < 0.01, ***P < 0.001 calculated with Kruskal-Wallis test (**B**, **D**, and **F**–**H**) or with Spearman’s rank correlation method (**I** and **J**). CTRL, healthy controls; COVID-19, patients with COVID-19; post–COVID-19, patients who recovered from COVID-19; FC, fold change.

In the evaluation of exhaustion markers, CD127^+^ and PD-1^+^ CD4^+^ T cell subpopulations were increased in patients who recovered from COVID-19 as compared with healthy controls, and they were at comparable levels to those observed in patients with COVID-19 (Figure 1, A–D), while 2B4^+^CD4^+^ T cell levels were similar to that of healthy controls but higher compared with patients with COVID-19 (Figure 1, E and F). Furthermore, an increased fraction of cells expressing LAG3, as well as a decreased proportion of cells marked by the expression of TIGIT, was observed in the CD4^+^ T cell population of patients who recovered from COVID-19 as compared with patients with COVID-19 and with healthy controls (Figure 1, G and H), suggesting the persistence of an abnormal exhaustion profile even after symptom remission. This was further confirmed in CD8^+^ T cells, in which several markers were upregulated in patients who recovered from COVID-19 as compared with healthy controls and/or compared with patients with COVID-19 (Table 2). Finally, transcriptomic profiling revealed an altered pattern of CD4^+^ and CD8^+^ T cell gene expression in patients who recovered from COVID-19 that particularly affected the CD4^+^ T cell subpopulation, with less dysregulation observed in CD8^+^ T cells (Figure 1, I and J). Indeed, downregulation of several proinflammatory genes, including *CSF1*, *LAT*, *LTA*, *BTLA*, *CD40LG*, *JAK1, TNFSF14*, and *TNFRSF9*, was evident in CD4^+^ T cells of patients who recovered from COVID-19 as compared with healthy controls (Figure 1I), with *LAT* and *BTLA* also downregulated in CD8^+^ T cells (Figure 1J). Furthermore, expression levels of genes controlling cell proliferation and apoptosis, such as *TGFB1*, *CDK4*, *TNFSF10*, and *TNFRSF10A*, were also found to be dysregulated in CD4^+^ T cells of patients who recovered from COVID-19 (Figure 1I). Overall, these results reveal unique features in the immunophenotype of patients who recovered from COVID-19.

### T cell overstimulation is associated with an abnormal secretome.

**Figure 2:**
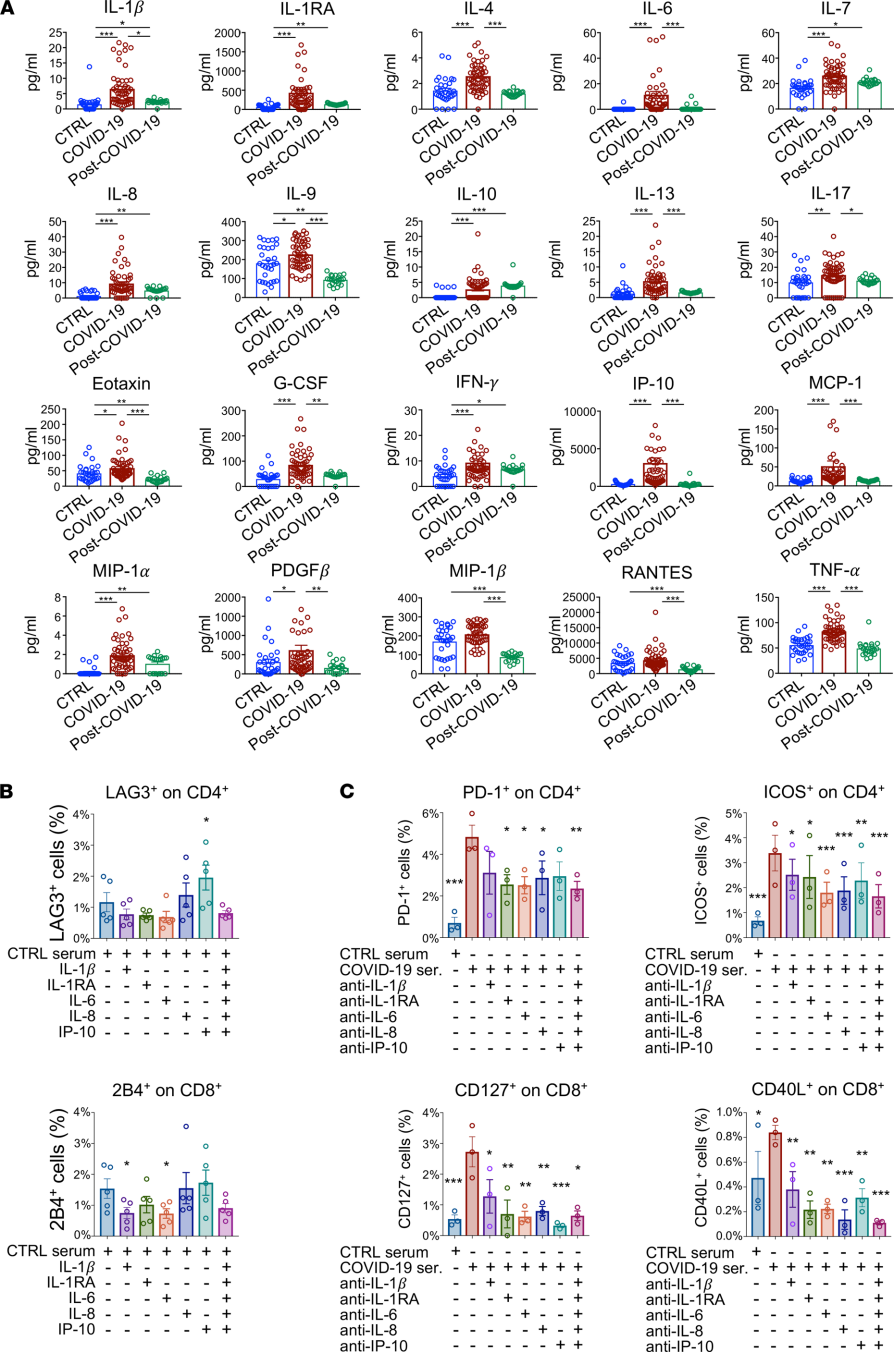
Cytokine profile and T cell exhaustion in patients who recovered from COVID-19 as compared with those with COVID-19 and with healthy controls. (**A**) Bar graphs depicting cytokine serum levels assessed by Luminex-based technology in patients with COVID-19 (*n* = 50), in those who recovered from COVID-19 (*n* = 20), and in healthy controls (*n* = 30). (**B**) Bar graphs depicting the percentage of LAG3^+^ cells in the CD4^+^ T cell population and of 2B4^+^ cells in CD8^+^ T cells assessed by flow cytometric analysis in PBMCs of healthy controls (*n* = 5) that were treated ex vivo with selected proinflammatory cytokines, either individually or as a pool. (**C**) Bar graph depicting percentage of PD-1^+^ and ICOS^+^ CD4^+^ T cells and of CD127^+^ and CD40L^+^ CD8^+^ T cells as assessed by flow cytometric analysis of PBMCs isolated from patients with COVID-19 (*n* = 5) that were exposed ex vivo to medium containing serum of patients with COVID-19 in the presence of blocking antibodies directed against IL-1β, IL-1RA, IL-6, IL-8, or IP-10, added either individually or as a pool. Data are reported as mean ± SEM unless otherwise reported. *P < 0.05, **P < 0.01, ***P < 0.001 calculated with Kruskal-Wallis test (**A**) or 1-way ANOVA (**B** and **C**). CTRL, healthy controls; COVID-19, patients with COVID-19; post–COVID-19, patients who recovered from COVID-19.

To characterize the cytokine signature of patients who recovered from COVID-19, we assessed plasma levels of a panel of 27 cytokines in subjects of the 3 groups using a multiplex Luminex-based system (Figure 2A and Supplemental Table 1). We found that plasma levels of cytokines IL-1β, IL-1RA, IL-7, IL-8, IL-10, IFN-γ, and MIP-1α were higher in patients who recovered from COVID-19 as compared with healthy controls and were at comparable levels to those observed in patients with COVID-19 (Figure 2A), indicating a failure to return to physiological cytokine levels after COVID-19. Intriguingly, levels of IL-9, eotaxin, MIP-1β, and RANTES in patients who recovered from COVID-19 were found to be the lowest among the 3 groups (Figure 2A), further indicating that cytokine levels are dysregulated in patients with COVID-19 after clinical symptom remission. To provide mechanistic insight into the relation between systemic release of proinflammatory factors and the abnormal immunological phenotype observed in T cells of patients with COVID-19, we exposed PBMCs isolated from healthy controls to several proinflammatory cytokines, which were increased in patients’ circulation during and after COVID-19. PBMCs were cultured for 48 hours in medium supplemented with human serum and containing recombinant IL-1β, IL-1RA, IL-6, IL-8, and IP-10 added either individually or as a pool; afterward, cells were collected for exhaustion and costimulatory marker analysis by flow cytometry. We found that recombinant IP-10 administration increased the fraction of CD4^+^ T cells that were positive for the LAG3 exhaustion marker, while a lower percentage of 2B4^+^ cytotoxic CD8^+^ T cells was detected in PBMCs cultured in medium containing recombinant IL-1β or IL-6 as compared with medium alone (Figure 2B and Supplemental Figure 2, A–C). To further investigate the role of these cytokines on T cell phenotype, we also exposed PBMCs isolated from patients with COVID-19 to medium containing their own serum in the presence of blocking antibodies directed against IL-1β, IL-1RA, IL-6, IL-8, or IP-10, added either individually or as a pool. We then assessed the resultant changes on expression of T cell exhaustion and activation markers by flow cytometry. PBMCs exposed to a pool of sera obtained from patients with COVID-19 increased the proportion of several exhaustion and costimulatory markers (Figure 2C and Supplemental Figure 3, A and B). Notably, we observed an overall reversal of the COVID-19 serum–induced increase in costimulatory and exhaustion marker expression on both CD4^+^ and CD8^+^ T cells following addition of blocking antibodies to cell cultures (Figure 2C and Supplemental Figure 3, A and B). In particular, the expression of PD-1 and ICOS on CD4^+^ T cells and that of CD127 and CD40L on CD8^+^ T cells showed a marked decrease when the majority of blocking antibodies were added, both individually and as a pool, to PBMCs cultured with serum obtained from patients with COVID-19 (Figure 2C and Supplemental Figure 3, A and B). Overall, these findings suggest that high levels of serum cytokines, at least in part, account for the T cell exhaustion observed in patients who recovered from COVID-19.

### T cells from patients who recovered from COVID-19 are dysfunctional.

**Figure 3:**
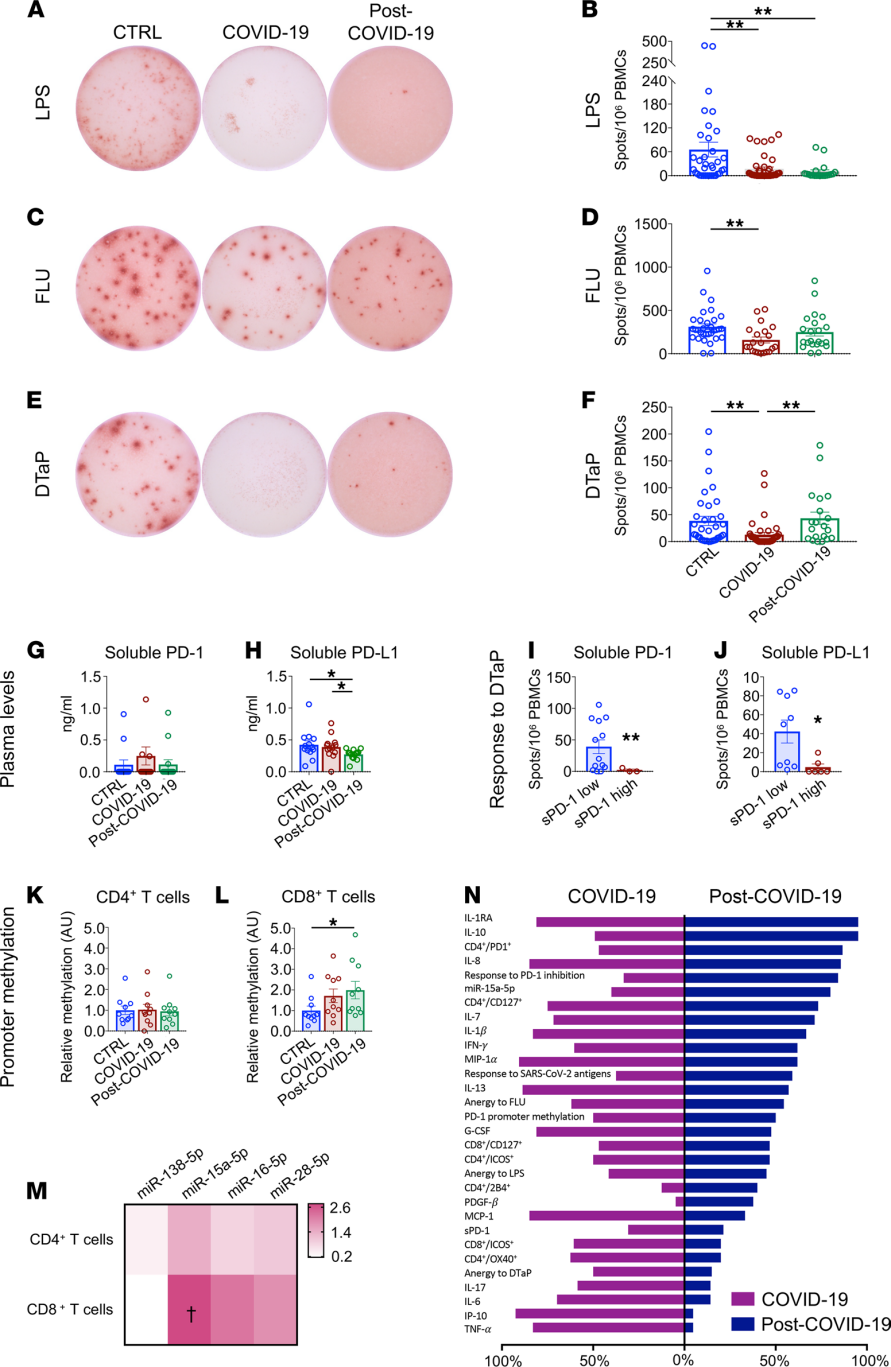
T cells from patients who recovered from COVID-19 are exhausted. (**A**–**F**) Representative images and bar graphs of ELISpot analysis depicting IFN-γ spots produced by PBMCs isolated from patients with COVID-19 (*n* = 40), from those who recovered from COVID-19 (*n* = 20), and from healthy controls (*n* = 30) following challenge with LPS (**A** and **B**), FLU (**C** and **D**), and DTaP (**E** and **F**). (**G** and **H**). Bar graphs depicting soluble PD-1 and soluble PD-L1 plasma levels in patients with COVID-19 (*n* = 13), in those who recovered from COVID-19 (*n* = 13), and in healthy controls (*n* = 14). (**I** and **J**) Bar graphs depicting the immune T cell response upon DTaP stimulation in patients with COVID-19 with high (above the median) versus low (below the median) levels of soluble PD-1 (**I**) or PD-L1 (**J**). (**K** and **L**) Relative levels of *PD-1* promoter DNA methylation in CD4^+^ (**K**) or CD8^+^ (**L**) T cells of patients with COVID-19 or after recovery as compared with healthy controls. (**M**) Heatmap showing color-coded relative levels of PD-1–targeting miR–138-5p, miR–15a-5p, miR–16-5p, and miR–28-5p miRNAs in CD4^+^ and CD8^+^ T cells of patients with COVID-19 and in those who recovered from COVID-19 (*n* = 10) normalized versus controls (*n* = 5). (**N**) Bar graph comparing the global immunological profiles of patients with COVID-19 after clinical symptom remission and during the acute phase of the disease. Each bar depicts the proportion of patients for which the value of the related factor is above the 75th percentile of the control group dataset. Data are expressed as mean ± SEM. *P < 0.05, **P < 0.01; ^†^*P* < 0.05 as compared with healthy controls, calculated with Kruskal-Wallis test (**B**, **D**, **F**–**H**, **K**, and **L**) or 2-tailed unpaired *t* test (**I**, **J**, and **M**). CTRL, healthy controls; COVID-19, patients with COVID-19; post–COVID-19, patients who recovered from COVID-19; DTaP, diphtheria-tetanus-pertussis vaccine; FLU, flu vaccine; sPD-1, soluble PD-1; sPD-L1, soluble PD-L1; FC, fold change; A.U., arbitrary units.

Given the persistent T cell activation/exhaustion observed in patients who recovered from COVID-19, we aimed to investigate the CD4^+^ T cell–dependent response following specific or nonspecific in vitro stimulation using an ex vivo IFN-γ–based ELISpot assay. PBMCs isolated from patients who recovered from COVID-19, from patients with COVID-19, and from healthy controls were exposed in vitro to LPS, diphtheria/tetanus/pertussis vaccine (DTaP), or quadrivalent flu vaccine (FLU), and the T cell–mediated response was evaluated in terms of number of IFN-γ–secreting cells, as indicated by the number of spots observed per 1 × 10^6^ plated PBMCs (Figure 3, A–F). Cells isolated from patients who recovered from COVID-19 showed a markedly decreased response to nonspecific stimulation as compared with healthy controls; this response resembled that of patients with COVID-19 (LPS; Figure 3, A and B) and represents an additional immunological feature that distinguishes patients who have recovered from COVID-19. Given the increase in PD-1–expressing T cells observed in patients who recovered from COVID-19 and the role of the PD-1/PD-L1 axis in exhaustion onset, we sought to further confirm dysregulation of the PD-1/PD-L1 pathway in COVID-19. We thus assessed plasma levels of soluble PD-1 (sPD-1) and soluble PD-1 ligand PD-L1 (sPD-L1) — as well as the expression of PD-1– and PD-L1–targeting miRNAs miR–138-5p, miR–15a-5p, miR–16-5p, and miR–28-5p in CD4^+^ and CD8^+^ T cells — and the extent of *PD1* promoter DNA methylation in CD4^+^/CD8^+^ T cells isolated from patients who recovered from COVID-19 as compared with those with COVID-19 and healthy controls (Figure 3, G–M). Interestingly, we found lower serum sPD-L1 levels in patients who recovered from COVID-19 as compared with both healthy controls and patients with COVID-19 (Figure 3H), suggesting an overstimulation of the PD-1/PD-L1 axis in these patients. This conclusion was further supported when we compared the PBMC-specific response to DTaP antigen stimulation in patients with higher (above the median) versus lower (below the median) plasma sPD-1 or sPD-L1 levels. High levels of both sPD-1 and sPD-L1 were associated with a marked decrease in the PBMC immune response to DTaP in patients who recovered from COVID-19 and patients with COVID-19 as compared with healthy controls (Figure 3, I and J). We next investigated the methylation status of a specific *PD1* promoter CpG site that is reported to control *PD1* gene expression in CD4^+^ and CD8^+^ T cells. Methylation-specific quantitative PCR (qPCR) results demonstrated an altered degree of *PD1* promoter methylation in CD8^+^ T cells — but not in CD4^+^ T cells — of subjects who recovered from COVID-19 as compared with healthy controls, confirming dysregulation of the PD-1/PD-L1 axis in patients who recovered from COVID-19 (Figure 3L). In CD8^+^ T cells — but not in CD4^+^ T cells — the PD-1 miRNet was also found to be altered, as shown by the higher expression of miR–15a-5p in patients who recovered from COVID-19 and in patients with COVID-19 as compared with healthy controls (Figure 3M). Overall, our results suggest an abnormal T cell phenotype and function of the immune response in patients who recovered from COVID-19 (Figure 3N), and this is at least partially due to a dysregulated PD-1/PD-L1 axis in T cells. Interestingly, these immune abnormalities were found to be associated with a persistence of dyspnea and several additional COVID-19–related symptoms at the time of recruitment (Table 3).

### In vitro PD-1 blockade restores T cell function.

**Figure 4:**
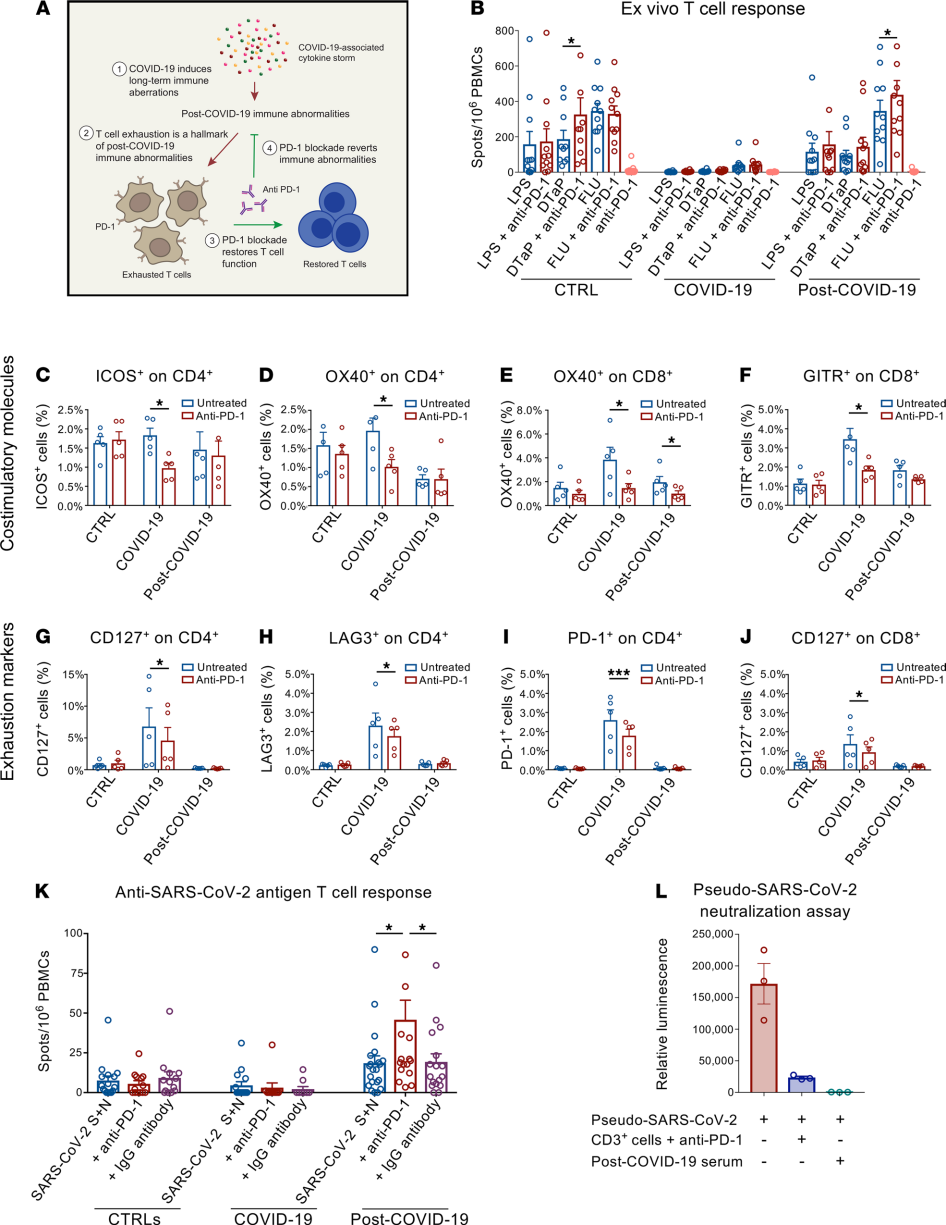
PD-1 blockade restores T cell function and the anti–SARS-CoV-2 antiviral T cell response in vitro. (**A**) Working hypothesis describing a PD-1 blockade–based strategy to reverse T cell exhaustion and restore the anti–SARS-CoV-2 immune response. (**B**) Bar graphs depicting the effect of PD-1 blockade on the number of IFN-γ spots produced by ELISpot analysis of PBMCs isolated from patients with COVID-19 (*n* = 40), from those who recovered from COVID-19 (*n* = 20) and from healthy controls (*n* = 35) following challenge with LPS, FLU, and DTaP, with or without anti–PD-1 blocking antibody. (**C**–**J**) Effect of PD-1 blockade on the proportion of the costimulatory markers ICOS and OX40 expressed by CD4^+^ T cells, GITR, and OX4 expressed by CD8^+^ T cells (**C**–**F**); exhaustion markers CD127, LAG3, PD-1 expressed by CD4^+^ T cells; and CD127 expressed by CD8^+^ T cells (**G**–**J**) in PBMCs isolated from patients who recovered from COVID-19 (*n* = 5) cultured either alone or in the presence of anti–PD-1 bocking antibody. (**K**) Effect of PD-1 blockade on the number of IFN-γ spots by ELISpot analysis using PBMCs isolated from patients with COVID-19 (*n* = 40), from those who recovered from COVID-19 (*n* = 20), and from healthy controls (*n* = 35) following challenge with spike and nucleocapsid SARS-CoV-2 peptides, with anti–PD-1 bocking antibody or with anti–human IgG antibody. (**L**) Efficient T lymphocyte–dependent neutralization of spike SARS-CoV-2 pseudotyped lentivirus by CD3^+^ T cells following PD-1 blockade as assessed by luminescence-based neutralization assay (*n* = 5). Serum of patients who recovered from COVID-19 was used as control. Data are expressed as mean ± SEM. *P < 0.05, ***P < 0.001 calculated with 2-tailed paired *t* test (**B**–**J**) or 1-way ANOVA (**K** and **L**). CTRL, healthy controls; COVID-19, patients with COVID-19; post–COVID-19, patients who recovered from COVID-19; DTaP, diphtheria-tetanus-pertussis vaccine; FLU, flu vaccine; SARS-CoV-2 S+N, SARS-CoV-2 spike and nucleocapsid peptide pool.

Based on these results, we hypothesized that PD-1 blockade could counteract post–COVID-19 immune abnormalities (Figure 4A). We therefore sought to determine whether PD-1 blockade, achieved by use of a clinically relevant anti–PD-1 blocking mAb, would revert the exhaustion status of T cells and restore their functional activity in response to specific and nonspecific stimulation. In an ELISpot ex vivo assay, we examined the function of T cells in patients who recovered from COVID-19 and in controls following challenge with LPS, DTaP, and FLU in the presence of anti–PD-1 mAb. FLU-stimulated PBMCs collected from patients who recovered from COVID-19 and cultured in the presence of anti–PD-1 displayed a significantly enhanced immune response as compared with FLU-only–stimulated PBMCs (Figure 4B). A similar pattern was observed when using PBMCs from healthy controls, in which cells cultured with anti–PD-1 mAb showed an increased response to DTaP stimulation as compared with DTaP-only–treated cells (Figure 4B). Given the increased expression of costimulatory and exhaustion T cell markers observed in patients who recovered from COVID-19, we next investigated the ability of PD-1 blockade to reverse this phenotype (Figure 4, C–J, and Supplemental Figure 4, A and B). Ex vivo anti–PD-1 blockade reversed the increase in the expression of the positive costimulatory marker OX40 in the CD8^+^ T cell fraction that was observed in patients who recovered from COVID-19 (Figure 4E). Moreover, PD-1 blockade in PBMCs isolated from patients with COVID-19 resulted in a decrease in the percentage of exhausted T cells (Figure 4, C–J). Taken together, our data indicate that, in T cells isolated from patients with COVID-19 upon PD-1 immune checkpoint blockade, T cell immune response and phenotype improve. We then examined, in an ex vivo assay, the T cell response to a pool of peptides derived from SARS-CoV-2 spike and nucleocapsid proteins, with or without the administration of anti–PD-1 blocking mAb, or in the presence of an anti–human IgG used as a negative control. The number of IFN-γ–producing cells was then quantified in an ELISpot assay. A sizeable T cell response against SARS-CoV-2 antigens at baseline was mounted only when PBMCs isolated from patients who recovered from COVID-19 were used (Figure 4K). Remarkably, the addition of anti–PD-1 mAb resulted in a 2-fold increase in the number of IFN-γ–producing cells as compared with cells treated with SARS-CoV-2 peptide only or compared with the negative control (Figure 4K). This finding confirms that PD-1 blockade is able to bolster the specific T cell immune response to SARS-CoV-2 antigens, thus reinstating their functional activation. We then verified if PD-1 blockade prevents T lymphocyte–mediated neutralization of SARS-CoV-2. To this aim, we developed a SARS-CoV-2 cell-based neutralization assay to indirectly assess the CD3^+^ T cell antiviral response. First, a SARS-CoV-2 spike pseudotyped lentivirus containing a luciferase reporter gene was exposed to CD3^+^ T cells isolated from patients who recovered from COVID-19 in the presence of anti–PD-1 blocking antibody. Residual pseudoviral particles were collected and used to infect SARS-CoV-2 infection–sensitive, ACE2-overexpressing 293T cells, and T lymphocyte neutralization activity was measured by comparing luciferase activity in infected cells (Figure 4L). The sera of patients who recovered from COVID-19 containing high titers of anti–SARS-CoV-2 IgG was used as a positive control. Lymphocyte-driven pseudovirus neutralization was observed when pseudoviral particles were exposed to CD3^+^ T cells of patients who recovered from COVID-19 upon PD-1 blockade, in a pattern comparable with that which was observed when sera of patients who recovered from COVID-19 was used (Figure 4L). We thus demonstrate that PD-1 blockade enhances the anti–SARS-CoV-2–specific immune response and reinforces PBMC-mediated SARS-CoV-2 antiviral activity.

## Discussion

The longitudinal dynamics of the immune response following COVID-19 has gathered attention, primarily because of its implications regarding the existence of long-term health concerns (7, 38–41). In this report, we have performed a comprehensive immunophenotypic and functional profiling of patients who have recovered from COVID-19. Our findings have enabled the identification and characterization of a panel of immunological parameters, the dysregulation of which persists after COVID-19–related symptom remission. Patients who recovered from COVID-19 show alterations in the proportion of immune cell subsets, including cytotoxic CD8^+^ T cells, as well as effector and effector memory T cells. The functional phenotype of T cells in these patients is also abnormal, displaying higher expression of several costimulatory and exhaustion T cell markers, including PD-1. These findings confirmed previous observations of a deranged immune profile in patients recovered from COVID-19 (15–17). According to our data, such functional imbalance is reflected at the transcriptional level by altered mRNA expression of several genes involved in T cell activation (*CSF1*, *LAT*, *LTA*, *CD40LG*, *JAK1*), modulation (*BTLA*), proliferation (*CDK4*), and apoptosis (*TGFB1*, *CDK4*, *TNFSF10*, and *TNFRSF10A*), highlighting the persistence of perturbation of T cell function after clinical remission from COVID-19. We also observed substantially higher amounts of several relevant cytokines, including IL-1β, IL-1RA, and IL-8, which were not restored to normal levels after resolution of the acute phase of the disease. The dysregulated functional phenotype observed in patients who recovered from COVID-19 may be at least partially linked to the abnormal systemic cytokine levels characterizing a cytokine storm, since PBMCs isolated from healthy controls and exposed to recombinant IL-1β, IL-6, or IP-10 are subject to altered expression of LAG3 and 2B4, while antibody-mediated blockade of different cytokines — including IL-1β, IL-1RA, IL-6, IL-8, and IP-10 — promotes reversal of the COVID-19 serum–induced increase in exhaustion and costimulatory markers observed in both CD4^+^ and CD8^+^ T cells. Overall, these findings reveal unique and abnormal features of the immunophenotype of patients who have recovered from COVID-19. T cells of patients who recovered from COVID-19 are unresponsive when challenged ex vivo with both specific (DTaP) and nonspecific (LPA) stimulations, and this failure to respond is accompanied by a higher fraction of PD-1–expressing T cells. Further analyses confirmed a dysfunctional PD-1/PD-L1 immune checkpoint axis in patients who recovered from COVID-19, as revealed by lower plasma levels of sPD-L1, as well as by increased expression of miR–15a-5p — a previously reported *PD1*-targeting miRNA with inhibitory effects — and by higher *PD1* promoter methylation occurring in CD8^+^ T cells as compared with healthy controls. These findings are consistent with the view that the PD-1/PD-L1 axis is overstimulated in T cells of patients who recovered from COVID-19. These data prompted us to test whether T cell exhaustion can be reversed and whether T cell function can be restored by PD-1 blockade. Indeed, the blockade of PD-1 enhanced the specific T cell–mediated response to a flu vaccine and reverted the observed overstimulation/exhaustion phenotype. Interestingly, we noticed that PD-1 blockade also increased the CD4^+^ T cell–mediated response to a pool of SARS-CoV-2 spike and nucleocapsid peptides, with no negative effect on lymphocyte-mediated SARS-CoV-2 antiviral activity. In patients with COVID-19, the T cell response is attenuated during the initial phases of the infection (8, 10, 11, 42, 43). While most current therapeutic options for treating patients with COVID-19 are directed to either contain replication of SARS-CoV-2 (21–25, 36) or to control inflammation (27–30, 44), a successful treatment may require a well-functioning immune system with proficient T cell function. The array of post–COVID-19 immune abnormalities we reported here may embody an immune weakness, which may favor COVID-19 recurrence and impair the ability of T cells to fight pathogens that can be reverted by PD-1 blockade. Anti–PD-1 blocking compounds are currently successfully used as immunotherapeutic tools to enhance the antitumor immune response in cancer patients (45) and are being investigated for alleviating and resolving chronic infections (46), as well as autoimmune diabetes (47–49). Future studies may support PD-1 blockade as a potential tool to correct the immune abnormalities persisting after remission from COVID-19 and restore full competence of the immune system. In summary, by using a broad phenotypic and functional immune characterization approach, we report here that patients who recovered from COVID-19 present with post–COVID-19 immunological abnormalities consisting of an aberrant immune cell and cytokine profile, as well as impaired T cell function, even months after hospital discharge. We also show that immune checkpoint PD-1 blockade reverts such immune abnormalities and restores a nearly normal T cell phenotype and function. Further studies performed on murine model would be ideal to ascertain the ability of in vivo PD-1 blockade to revert phenotypic and functional immune alterations induced by SARS-CoV-2 infection that persist after recovery from COVID-19.

## Methods

Supplemental Methods are available online with this article.

### Patients.

Consecutively admitted patients with COVID-19 or patients who recovered from COVID-19 infection admitted for SARS-CoV-2 acute infection at the Infectious Disease and Respiratory Division of ASST FBF-Sacco in Milan, Italy, from March 31, 2020, to October 2, 2020, were enrolled and compared with a group of healthy control subjects. SARS-CoV-2 infection of all enrolled patients was confirmed by viral PCR of nasal and pharyngeal swab specimens collected during the acute phase of the infection, according to WHO guidance. Patient baseline clinical score was determined according to a modified ordinal score based on 7 major points as previously reported (31). Baseline demographic distributions and clinical, laboratory, management, and outcome data were abstracted from patient electronic medical reports. Immunophenotyping was performed by flow cytometry using antibodies listed in Supplemental Table 2.

### ELISpot assay.

An ELISpot assay was used to measure the number of IFN-γ–producing cells according to the manufacturer’s protocol (BD Biosciences) as previously shown by our group (50). For testing nonspecific ex vivo T cell responses, 3 × 10^5^ PBMCs isolated from healthy control subjects, from patients with COVID-19, and from patients who recovered from COVID-19 were cultured for 48 hours in RPMI 1640 10% FBS in the presence of LPS (1 μg/mL; Merck), FLU, or a DTaP, with or without the addition of an anti–PD-1 blocking antibody (pembrolizumab, 5 μg/mL). At day 2 after stimulation, cells were collected and plated on a human IFN-γ ELISpot assay plate, and spots were counted using an ImmunoSpot Reader (CTL Europe GmbH). The specific response to SARS-CoV-2 antigens was tested by culturing 3 × 10^5^ PBMCs isolated from healthy control subjects, patients with COVID-19, and patients who recovered from COVID-19 for 48 hours in RPMI 1640 10% FBS in the presence of pooled peptides derived from SARS-CoV-2 spike and nucleocapsid proteins (PR-nCoV-1, PR-nCoV-3; Novatein Biosciences) (1 μg/mL) with or without the addition of the anti–PD-1 blocking antibody pembrolizumab (Keytruda, MSD) (5 μg/mL) or of an anti–human IgG antibody used as a negative control (clone 83.8F9, LifeSpan Bioscience) (5 μg/mL). At day 2 after stimulation, cells were collected and plated using the human IFN-γ ELISpot assay and processed as previously described.

### Pseudo–SARS-CoV-2 neutralization assay.

Spike SARS-CoV-2 pseudotyped lentiviruses containing a luciferase reporter (BPS Bioscience) were used in our modified neutralization assay. Briefly, 5 μL of SARS-CoV-2 pseudotyped lentivirus were incubated with 5 × 10^5^ PBMCs from patients who recovered from COVID-19 with the addition of anti–PD-1 blocking antibody. Cells were cultured in RPMI 1640 in a 96-well white clear-bottom assay plate and incubated for 12 hours at 37°C. SARS-CoV-2 pseudotyped lentivirus incubated with serum collected from patients who recovered from COVID-19 served as a positive control. After incubation, supernatants were collected from every condition and used for infection of ACE2-overexpressing 293T cells (BPS Bioscience); they were then further incubated for 48 hours in 5% CO_2_ at 37°C. Luminescence, which is correlated to the luciferase activity on 293T cells, was quantified using the One-step Luciferase Assay System as recommended by the manufacturer on a GloMAX luminometer (both from Promega).

### Statistics.

Prism version 7.0 (GraphPad) was used for statistical analysis and graphical representation of data. The sample distribution was determined by the Shapiro-Wilk normality test. One-way ANOVA, 2-tailed *t* test, χ^2^ test, or nonparametric Kruskal-Wallis and Mann-Whitney *U* tests were performed where appropriate and according to data distribution. All data are presented as mean ± SEM, with *P* values less than 0.05 considered as significant.

### Study approval.

All samples were obtained from patients and healthy controls after provision of informed consent and in accordance with the Ethical Research Committee of the Sacco Hospital (Comitato Etico Milano Area 1), which granted the approval of the present study.

## Author contributions

CL designed and performed experiments, analyzed data, and wrote and edited the paper; AA, FD, and MBN designed and performed research, analyzed the data, and edited the paper; EA, VU, AM, AJS, EI, SDM, LL, CV, and MB collected and analyzed data; DR, A. Rossi, IP, LM, MEL, AMB, SA, and A. Riva assisted with sample collection and data analysis; JY, BEE, PS, GVZ, SR, and MG coordinated research and edited the paper; PF conceived the idea, designed the study, and wrote and edited the paper. All authors were given full access to all data presented in this study and are responsible for the integrity of the data and accuracy of the data analysis. All authors have given their permission for submission of this manuscript.

## Supplementary Material

Supplemental data

## Figures and Tables

**Table 1:**
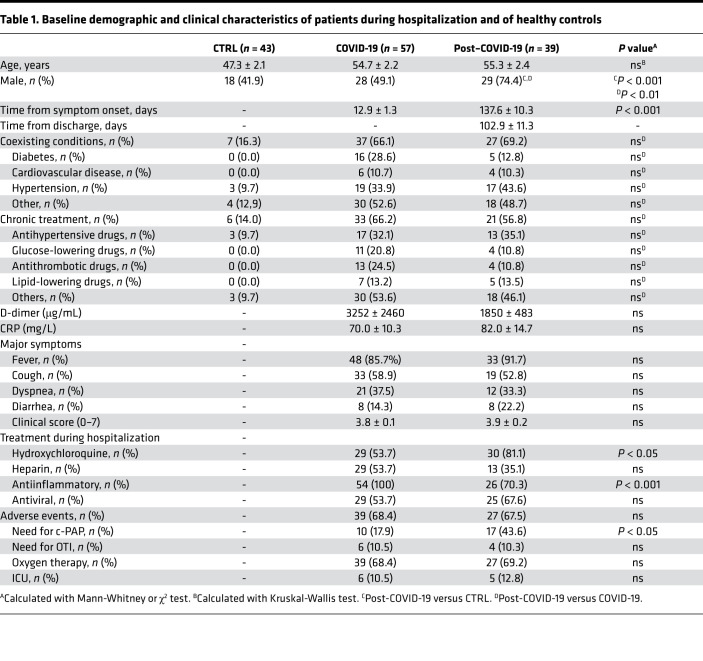
Baseline demographic and clinical characteristics of patients during hospitalization and of healthy controls

**Table 2:**
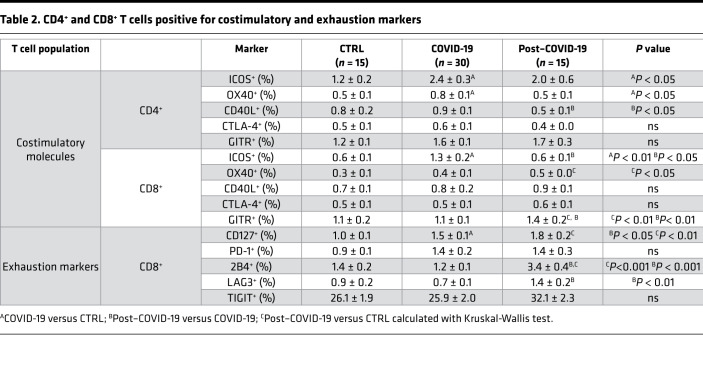
CD4^+^ and CD8^+^ T cells positive for costimulatory and exhaustion markers

**Table 3:**
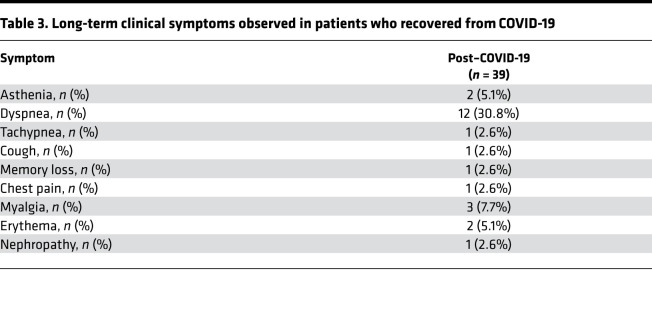
Long-term clinical symptoms observed in patients who recovered from COVID-19

## Version 1 11/16/2021

In-Press Preview

## Version 2 12/22/2021

Electronic publication
