# Supplementary material for: PD-1 blockade counteracts post–COVID-19 immune abnormalities and stimulates the anti–SARS-CoV-2 immune response
Source: JCI Insight. 2021 Dec 22;6(24):e146701. doi: 10.1172/jci.insight.146701 (PMC8783674; doi:10.1172/jci.insight.146701)
Supplement: Supplemental data [file jciinsight-6-146701-s040.pdf]

Title: PD-1 blockade counteracts post-COVID-19 immune abnormalities and stimulates the anti-SARS-CoV-2 immune response

This Supplemental Information file includes:

- **Supplemental Materials and Methods**
- **Supplemental Data:**
  - **Supplemental Figures S1-S4**
  - **Supplemental Tables S1-S2**
- **Supplemental References**

Supplemental Materials and Methods

Patient selection

Adult patients included in this study were divided into 3 groups. The first group was comprised of patients hospitalized at the ASST Fatebenefratelli-Sacco Hospital of Milan, Italy for COVID-19 in the period between March 31, 2020 and October 2, 2020. A second group included patients who had recovered from COVID-19 and were undergoing clinical follow-up for a period of 2-6 months after discharge from the hospital. A group of healthy controls with no previous diagnosis of COVID-19 and with a negative test for anti-SARS-CoV-2 serum IgM and IgG antibodies was also included in the study. Informed consent was obtained from all enrolled subjects before recruitment. This study received approval from the Institutional Review Board of Luigi Sacco Hospital, Milan.

Peripheral blood mononuclear cell purification

Peripheral blood mononuclear cells (PBMCs) were purified from 20 ml blood samples collected from patients with COVID-19, from patients who recovered from COVID-19 and from healthy controls by gradient centrifugation. Cells were then frozen in 2-5 x 10^6 cells aliquots and stored in liquid nitrogen vapor.

Flow cytometric analysis

In order to immunologically characterize patient cell subpopulations, frozen PBMCs were thawed and allowed to rest overnight in complete medium containing RPMI 1640 (Life Technologies, Carlsbad, CA, USA) supplemented with 10% FBS (Capricorn Scientific, Ebsdorfergrund, Germany), 2 mM L-glutamine and 100 U/ml penicillin (Life Technologies). Cells were then harvested and washed with FACS buffer (PBS supplemented with 1% BSA and 0.5 mM EDTA) and stained with anti-human CD45, CD3, CD4, CD8, CD19, CD14, CD45RO, CD45RA, CD62L, CD127, CD25, and FoxP3 antibodies for immune cell subset characterization of PBMCs, or with anti-human CD4, CD8, CD127, PD-1, LAG3, 2B4, TIGIT,

OX40, ICOS, CTLA-4, CD40-L and GITR antibodies for the analysis of exhaustion and costimulatory molecule markers on PBMCs. Antibodies used were purchased from BD Biosciences, San Jose, CA, USA and are detailed in Supplemental Table S1. Sample acquisition was performed on a FACSCelesta™ flow cytometer system equipped with BD FACSDiva software (BD Biosciences), and data were analyzed using FlowJo software, version 10 (TreeStar, Ashland, OR).

Peripheral cytokine profiling

Circulating plasma cytokine levels were measured in patients with COVID-19, in patients recovered from COVID-19 and in healthy controls using a Luminex®-based Bio-Plex Pro Human Cytokine 27-plex Assay (Bio-Rad Laboratories, Hercules, CA, USA). The panel of analytes included IL-1β, IL-1RA, IL-2, IL-4, IL-5, IL-6, IL-7, IL-8, IL-9, IL-10, IL-13, IL-15, IL-17, G-CSF, IFN-γ, IP-10, MCP-1, MIP-1α, TNF-α, IL-12P, eotaxin, FGF-2, GM-CSF, MIP1β, PDGF, RANTES and VEGF. Plasma of recruited individuals was collected and stored at −80°C until the time of analysis. On the day of assessment, plasma samples were gradually thawed and spun at 10,000 x g for 10 minutes. 50 uL of each 4-fold diluted plasma sample were then added to fluorescence-labeled microbeads, processed following the manufacturer's instructions and then run in a Bio-Plex 200 Multiplex Immunoassay System (Bio-Rad). Fluorescence results were analyzed using the in-house Bio-Plex Manager software, version 6.0 (all from Bio-Rad).

ELISpot assay

An ELISpot assay was performed to assess the T cell immune response to different stimuli using PBMCs isolated from patients with COVID-19, from patients who recovered from COVID-19 and from healthy controls. Assessment of non-specific and antigen-mediated ex vivo T cell response was performed on $3x10^5$ PBMCs cultured for 48h in RPMI 1640 medium supplemented with 10% FBS in the presence of 1 μg/ml lipopolysaccharide (LPS, Merck

KGaA, Darmstadt, Germany), influenza vaccine 1% v/v (FLU, Vaxigrip, Sanofi Pasteur, Paris, France), or a trivalent diphtheria, tetanus and pertussis vaccine 1% v/v (DTaP, Polio boostrix, GSK, Brentford, UK), either individually or with the addition of anti-human PD-1 monoclonal antibody (mAb) pembrolizumab (Keytruda, MSD, Kenilworth, NJ, USA) at the working concentration of 5 μg/ml. Cells were then collected and plated on immobilon-P white plates (Merck) precoated with an anti-IFN-γ capture antibody (Ab) (BD Biosciences). After 24 hours of incubation at 37°C, cells were discarded, and a biotinylated anti-IFN-γ detection Ab (BD Biosciences) was added to each well of the plate, which was then incubated overnight at 4° C. IFN-γ release was detected by adding horseradish peroxidase-conjugated streptavidin and 3-amino-9-ethylcarbazole (AEC) substrate (BD Biosciences) as specified in the manufacturer's instructions. Finally, the number of spots generated by IFN-γ-releasing cells were counted using an ImmunoSpot Reader (CTL Europe GmbH, Bonn, Germany). The number of spots observed in unstimulated cells were considered background and were subtracted from the number of spots observed in stimulated cells. Values are presented as number of spots normalized to $10x10^6$ PBMCs. In order to test for the anti-SARS-CoV-2 specific ex vivo T cell response, $3x10^5$ PBMCs freshly isolated from patients and healthy controls were cultured for 48h in RPMI 1640 plus 10% FBS complete medium in the presence of a pool of SARS-Cov-2 spike and nucleocapsid proteins (Novatein Biosciences, Hudson, MA, USA; 0.5 μg/ml each) for 48 hours, with or without pembrolizumab (5 μg/ml) and using an anti-human IgG antibody as a negative control (Yumab, Braunschweig, Germany). Cells were then collected and processed as described above.

Cytokine treatment of PBMCs in vitro

The exhaustion and costimulatory profiles of PBMCs isolated from healthy controls were analyzed following exposure to the cytokines IL-1β, IL-1RA, IL-6, IL-8 and IP-10. $1x10^6$ PBMCs isolated from 5 healthy individuals were cultured in 96-well plates in RPMI 1640

medium containing 10% human serum comprised of a pool of 5 healthy individuals' heat-inactivated human sera. Human recombinant IL-1β (15 pg/ml), IL-6 (10 ng/ml), IL-8 (25 pg/ml) and IP-10 (1 ng/ml) (all from R&D, Minneapolis, Min, USA) were added to the culture either individually or as a pool. The cytokine concentration in each treatment was chosen according to the average plasma concentration levels observed in patients with COVID-19. Each treatment was performed in 5 replicates using PBMCs obtained from 5 different healthy controls. After 24 hours of incubation, cells were recovered and prepared for flow cytometric analysis using a panel of costimulatory molecules and exhaustion markers as described above.

In vitro cytokine inhibition

To investigate the immunological effect of the blockade of cytokines that are overrepresented in the serum of patients with COVID-19, PBMCs were isolated from 3 patients with COVID-19 and then cultured in complete medium containing a pool of heat-inactivated sera obtained from 5 patients with COVID-19, selected on the basis of their high serum cytokine levels. Then, antibodies directed against IL-1β, IL-1RA, IL-6, IL-8, IP-10, IL-7, and TNF-α (each at 10 µg/ml) and IL-1RA (20 µg/ml) (all from R&D) were added to cultured PBMCs, either individually or as a pool. In parallel, PBMCs were also cultured with a pool of heat inactivated sera obtained from 5 healthy individuals. After 24 hours of incubation, cells were recovered and prepared for flow cytometric analysis of a panel of costimulatory molecules and exhaustion markers, as detailed above.

Quantitative RT-qPCR analysis

The T cell exhaustion profile of $CD4^+$ and $CD8^+$ T cells was investigated at the transcriptional level using RNA extracted from $CD4^+$ and $CD8^+$ cells isolated by magnetic bead-based positive selection kits (Miltenyi Biotec, Bergisch Gladbach, Germany), according to the manufacturer's instructions. RNA was extracted from each cell population using the Direct-zol RNA Miniprep kit (Zymo Research, Irvin, CA, USA), samples were then quality-checked for RNA integrity

by agarose gel electrophoresis, and RNA was then quantified and quality-checked for the presence of impurities using a Multiskan Go spectrophotometer (Thermo Fisher Scientific, Waltham, MA, USA). 200 ng RNA was reverse-transcribed using a Quantinova Reverse Transcription Kit (Qiagen, Hilden, Germany) following the manufacturer's instructions. cDNA for each sample was then analyzed by quantitative real-time PCR analysis using a SYBR green-based Quantinova LNA Human T Cell Anergy & Immune Tolerance focus panel (Qiagen) according to the manufacturer's instructions. Relative quantification of each gene in the panel was calculated by an in house-developed and delta-delta-Ct method-based Qiagen online tool (www.qiagen.com/geneglobe), using averaged levels of *ACTB*, *B2M*, *GAPDH*, *HPRT1* and *RPLP0* genes for data normalization. For heatmap generation, values for each gene were normalized to the mean across groups, log-transformed and then mean-polished, normalized, and clustered using the Spearman rank correlation method with Cluster 3.0 software (http://bonsai.hgc.jp/).

Soluble PD-1 and soluble PD-L1 assessment

Soluble PD-1 (sPD-1) and PD-L1 (sPD-L1) plasma levels for patients with COVID-19, patients who recovered from COVID-19 and healthy controls were measured quantitatively by an ELISA immunoassay. Levels of sPD-1 and sPD-L1 were measured using 100 µl aliquots of plasma with two commercially available sandwich enzyme-linked immunosorbent assays (Cloud-Clone Corp., Houston, TX, USA) according to the manufacturer's recommendations. The minimum detectable level for both sPD-1 and sPD-L1 was 0.156 ng/ml; samples for which signal was below the detectable level were assigned a value of 0 ng/ml.

Analysis of* PD1*-targeting miRNAs

Four *PD1*-targeting miRNAs (miR-138-5p, miR-15a-5p, miR-16-5p, miR-28-5p) were selected for their role in directly controlling *PD1* expression, according to previously published reports (1-3). $CD4^+$ and $CD8^+$ T cells were isolated from PBMCs by immunomagnetic cell separation

methods as described above, and total RNA was extracted from isolated cells using TRIzol reagent (Merck). Selected miRNAs were reverse-transcribed, and their levels were quantified by qPCR using a TaqMan MicroRNA Reverse Transcription Kit and the TaqMan MicroRNA Assays 002284 (miR-138-5p), 000389 (miR-15a-5p), 000391 (miR-16-5p) and 000411 (miR-28-5p) on a QuantStudio 7 Flex qPCR system (all from Thermo Fisher Scientific) according to the manufacturer's instructions. Data were analyzed by the delta-delta-C_t method using U6 small RNA as endogenous control.

PD1 *promoter methylation analysis*

PD1 promoter methylation status in $CD4^+$ and $CD8^+$ T cells was assessed by a bisulfite conversion-based quantitative real-time PCR method. $CD4^+$ and $CD8^+$ T cells were isolated from PBMCs by immunomagnetic cell separation methods as described above. DNA was extracted and bisulfite-converted from 50,000 cells of each sample using the EZ DNA Methylation-Direct Kit (Zymo Research). 25 ng converted DNA was then used as template in a SYBR green-based qPCR on a QuantStudio 7 Flex qPCR system (Thermo Fisher Scientific) using PowerUP SYBR Green reagent (Thermo Fisher Scientific) and primers targeting a *PD1* promoter CpG methylation site located in chr2:241859917-241859918 (hg38 assembly) that is known to affect PD-1 function/expression (4, 5). Relative DNA methylation of the *PD1* locus was determined by comparison to total DNA as determined via *ACTB* reference using primers targeting a DNA region devoid of CpG methylation sites. Primer sequences were designed using the MethPrimer online tool (6) and were as follows: methylated *PD1* forward, 5'-TATATTTTTGAGATTCGGGA-3'; methylated *PD1* reverse, 5'-GCCTAACAACTAACGC GACT-3'; *ACTB* forward, 5'-GTGATGGAGGAGGTTTAGTAAGTT-3'; *ACTB* reverse, 5'-CCAATAAAACCTACTCCTCCCTTAA-3'. *PD1* promoter methylation data obtained from patients with COVID-19 and from those who recovered from COVID-19 are presented as relative to the fraction of DNA methylation found in controls.

Supplemental Figures

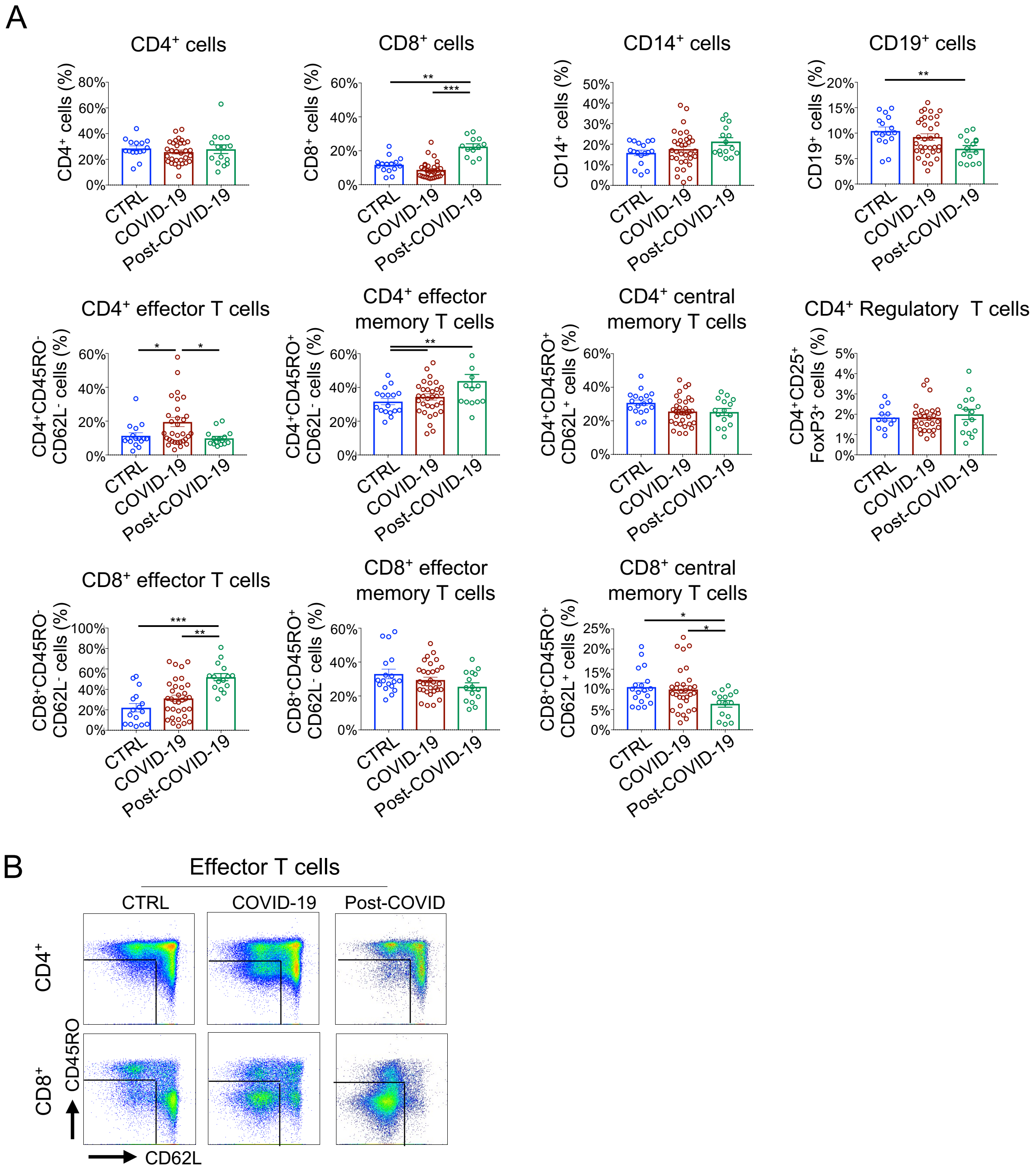

Supplemental Figure S1. Immunophenotyping analysis of patients with COVID-19 and in patients who recovered from COVID-19 as compared to healthy controls. (A). Bar graphs depicting the fraction of the primary PBMC subpopulations in patients with COVID-19 (n=30), in patients who recovered from COVID-19 (n=15) and in healthy controls (n=15). **(B)**. Representative dot plot depicting the percentage of effector (CD62L$^-$, CD45RO$^-$) T cells in the

same subjects. Data are reported as mean ± standard error (SEM) unless otherwise reported. *p<0.05, **p<0.01, ***p<0.001 calculated with Kruskal-Wallis test.

Abbreviations: CTRL, healthy controls; COVID-19, patients with COVID-19; Post-COVID-19, patients who recovered from COVID-19.

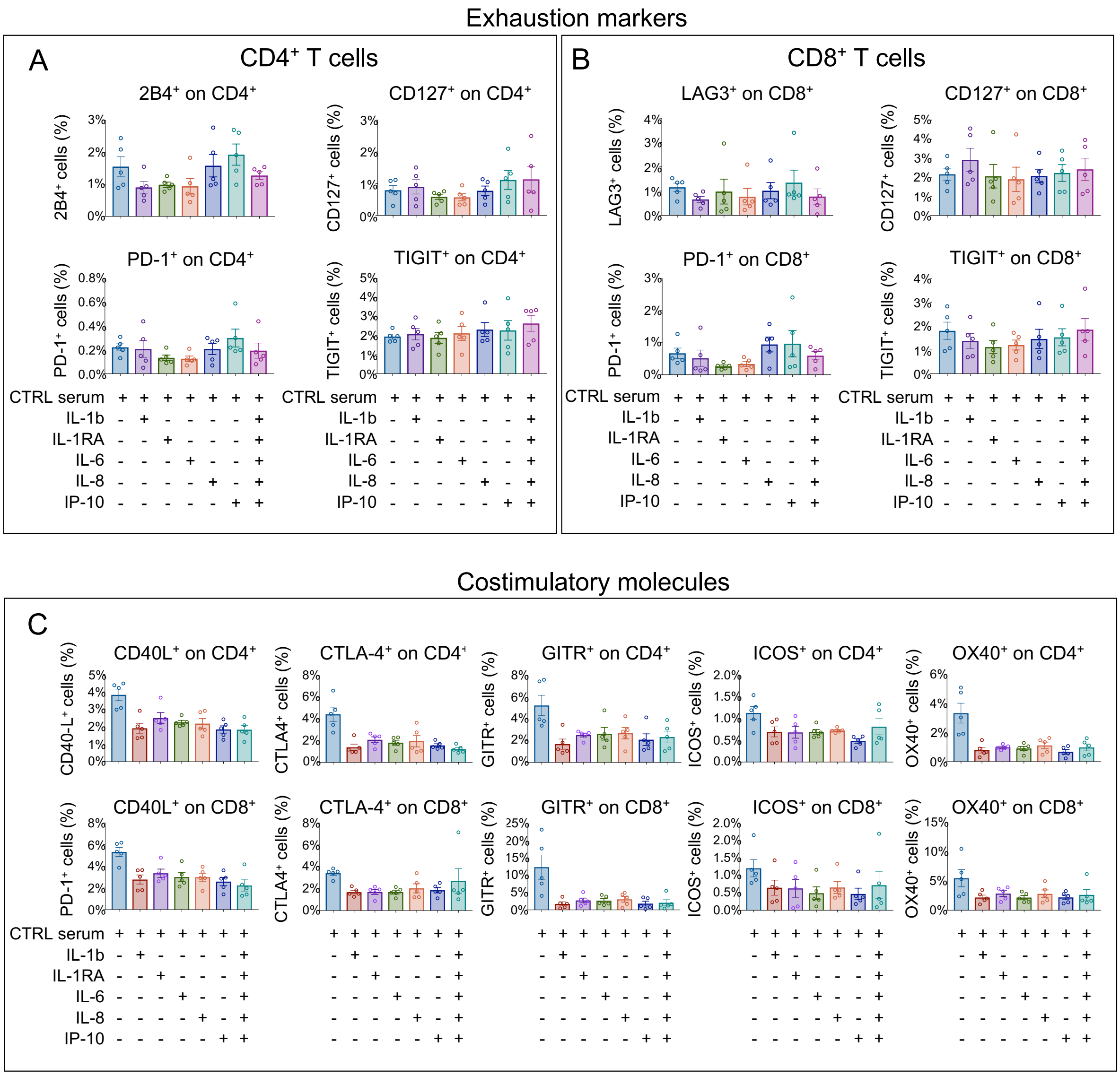

Supplemental Figure S2. T cell exhaustion and costimulatory marker analysis following cytokine administration to PBMCs obtained from healthy controls. (A-B). Bar graphs depicting the percentage of $CD4^+$ (**A**) and $CD8^+$ (**B**) T cells expressing the exhaustion markers included in the study as assessed by flow cytometric analysis in PBMCs of healthy controls (n=5) that were treated ex vivo with selected proinflammatory cytokines, either individually or as a pool. **(C).** Bar graphs depicting the percentage of $CD4^+$ and $CD8^+$ T cells expressing costimulatory markers as assessed by flow cytometric analysis of PBMCs obtained from patients with COVID-19 (n=5) and treated as described above. Data are reported as mean ±

standard error (SEM) unless otherwise reported. One-way ANOVA was used for data comparison.

Abbreviations: CTRL, healthy controls; COVID-19, patients with COVID-19; Post-COVID-19, patients who recovered from COVID-19; PBMCs, peripheral blood mononuclear cells.

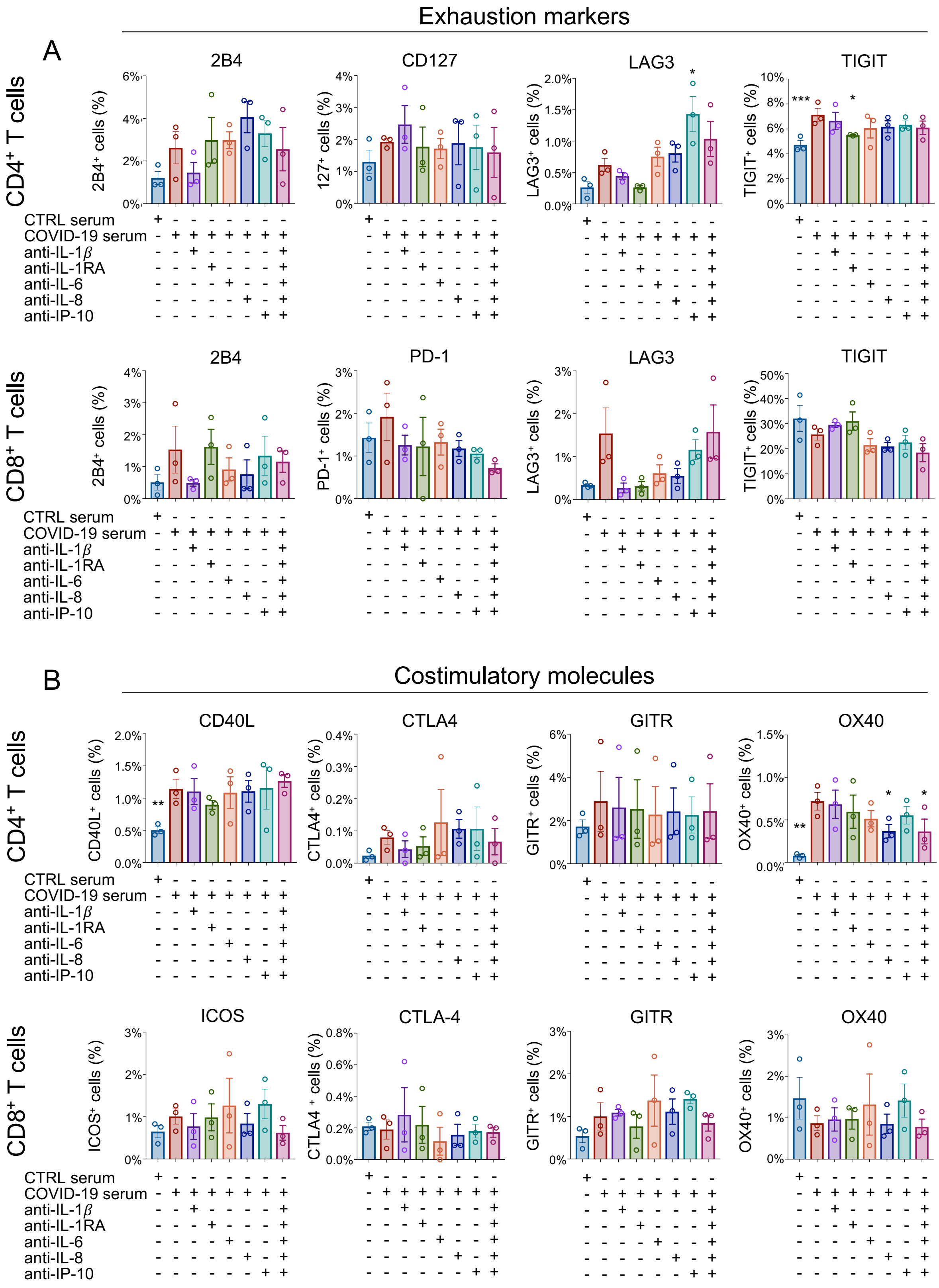

Supplemental Figure S3. Effect of in vitro cytokine inhibition on T cell immunophenotype in PBMCs cultured with serum obtained from patients with COVID-19. (A-B). Bar graphs depicting the percentage of CD4^{+} and CD8^{+} T cells expressing exhaustion (**A**) or costimulatory

markers (**B**) as assessed by flow cytometric analysis of PBMCs obtained from patients with COVID-19 (n=5) that were exposed ex vivo to a medium containing serum of patients with COVID-19 in the presence of blocking antibodies directed against IL-1β, IL-1RA, IL-6, IL-8 or IP-10, added either individually or as a pool. Data are reported as mean ± standard error (SEM) unless otherwise reported. *$p<0.05$, ***$p<0.001$ as compared to COVID-19 serum-treated PBMCs calculated with one-way ANOVA.

Abbreviations: CTRL, healthy controls; COVID-19, patients with COVID-19; Post-COVID-19, patients who recovered from COVID-19; PBMCs, peripheral blood mononuclear cells.

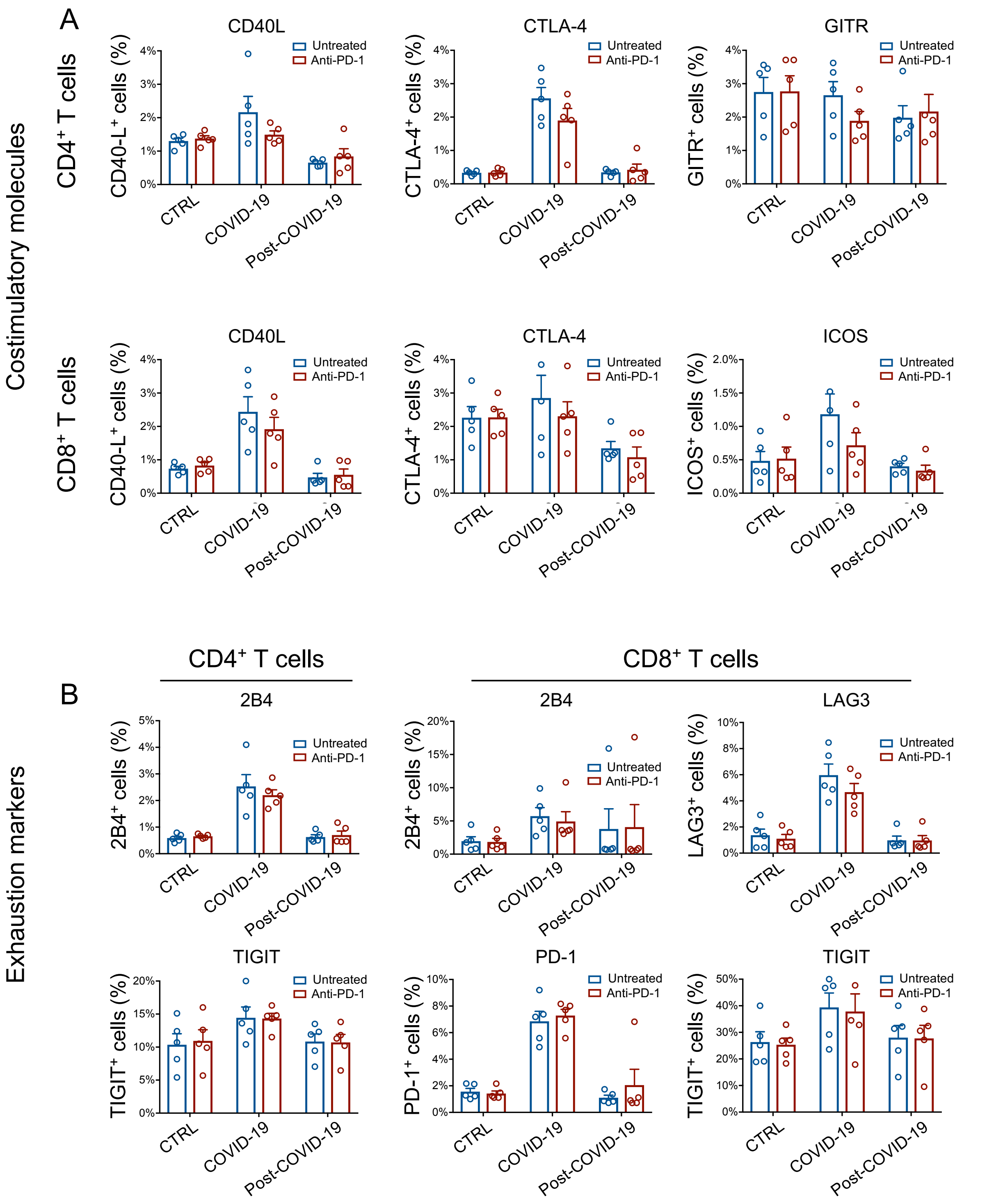

Supplemental Figure S4. Effect of ex vivo PD-1 blockade on T cell immunophenotype. (A-B). Bar graphs showing the effect of PD-1 blockade on the proportion of costimulatory (**A**) and exhaustion markers (**B**) on CD4$^+$ and CD8$^+$ T cells as assessed by flow cytometric analysis of PBMCs obtained from patients who recovered from COVID-19 (n=5), cultured either alone

or in the presence of anti-PD-1 bocking antibody. Data are reported as mean ± standard error (SEM) unless otherwise reported. Two-sided paired t test was used for data comparison.

Abbreviations: CTRL, healthy controls; COVID-19, patients with COVID-19; Post-COVID-19, patients who recovered from COVID-19; PBMCs, peripheral blood mononuclear cells.

Supplemental Tables

Supplemental Table S1. Details of antibodies used in the study.

Antibody	*Clone*	*Fluorophore*	*Company*	*Catalogue no.*	*Dilution factor*
CD3	UCHT1	BUV737	BD Biosciences	564307	1:100
CD45	HI30	BUV395	BD Biosciences	563792	1:100
CD4	SK3	BB700	BD Biosciences	566392	1:100
CD8	SK1	BV605	BD Biosciences	564116	1:100
CD25	2A3	BV421	BD Biosciences	564033	1:100
CD45RO	UCHL1	BV510	BD Biosciences	563215	1:100
CD62L	SK11	BV711	BD Biosciences	565040	1:100
CD127	HIL-7R-M21	BV786	BD Biosciences	563324	1:100
CD40-L	TRAP1	PE	BD Biosciences	555700	1:100
GITR	V27-580	BV480	BD Biosciences	747658	1:100
ICOS	DX29	BV711	BD Biosciences	563833	1:100
OX40	ACT35	BV650	BD Biosciences	563658	1:100
CTLA-4	BNI3	PE	BD Biosciences	562742	1:100
LAG-3	T47-530	BV480	BD Biosciences	746609	1:100
2B4	2-69	BV421	BD Biosciences	565750	1:100
TIGIT	741182	BV711	BD Biosciences	747839	1:100
PD-1	EH12.1	PE	BD Biosciences	562516	1:100
FoxP3	259D/C7	PE	BD Biosciences	560046	1:100
CD19	SJ25C1	BV650	BD Biosciences	563226	1:100
CD14	MφP9	PE	BD Biosciences	562691	1:100

Supplemental Table S2. FGF2 plasma levels in patients who recovered from COVID-19 as compared to those with COVID-19 and to healthy controls.

	CTRL (n=30)	*COVID-19 (n=50)*	*Post-COVID-19 (n=20)*	*p value*
FGF2 (pg/ml)	25.5 ± 2.3	36.2 ± 1.9§	26.8 ± 0.7¥	§p<0.001<br>¥p<0.01

Abbreviations. CTRL, healthy controls; COVID-19, patients with COVID-19; Post-COVID-19, patients who recovered from COVID-19; FGF2, fibroblast growth factor 2; (§): COVID-19 vs. CTRL; (¥): Post-COVID-19 vs COVID-19 calculated with Kruskal-Wallis test.

Supplemental references

1. Huemer F, et al. miRNA-Based Therapeutics in the Era of Immune-Checkpoint Inhibitors. *Pharmaceuticals (Basel).* 2021;14(2).
2. Jia X, et al. Deficiency of miR-15a/16 upregulates NKG2D in CD8(+) T cells to exacerbate dextran sulfate sodium-induced colitis. *Biochem Biophys Res Commun.* 2021;554:114-22.
3. Ding L, et al. Regulation of PD-1/PD-L1 Pathway in Cancer by Noncoding RNAs. *Pathol Oncol Res.* 2020;26(2):651-63.
4. Goltz D, et al. Promoter methylation of the immune checkpoint receptor PD-1 (PDCD1) is an independent prognostic biomarker for biochemical recurrence-free survival in prostate cancer patients following radical prostatectomy. *Oncoimmunology.* 2016;5(10):e1221555.
5. Rover LK, et al. PD-1 (PDCD1) Promoter Methylation Is a Prognostic Factor in Patients With Diffuse Lower-Grade Gliomas Harboring Isocitrate Dehydrogenase (IDH) Mutations. *EBioMedicine.* 2018;28:97-104.
6. Li LC, and Dahiya R. MethPrimer: designing primers for methylation PCRs. *Bioinformatics.* 2002;18(11):1427-31.
